# Effects of probiotic supplementation on islet β-cell function in subjects with glucose metabolism disorders: a meta-analysis

**DOI:** 10.3389/fnut.2025.1668470

**Published:** 2025-10-02

**Authors:** Mengyue Xiang, Xingkang Sa, Zemin Tuo, Jiwen Bian, Peng Wang, Xinming Zhang

**Affiliations:** ^1^Department of General Chinese Medicine, Shandong University of Traditional Chinese Medicine, Jinan, China; ^2^Department of Oncology, Qingdao Public Health Clinical Center, Qingdao, China; ^3^Department of General Practice, Weifang Hospital of Traditional Chinese Medicine, Shandong Second Medical University, Weifang, China; ^4^Department of General Surgery, Qingdao Traditional Chinese Medicine Hospital (Qingdao Hiser Hospital Affiliated to Qingdao University), Qingdao, China

**Keywords:** diabetes mellitus, gestational diabetes mellitus, prediabetes, HOMA-β, probiotics

## Abstract

**Background:**

Islet β-cell dysfunction is central to the pathophysiology of glucose metabolism disorders. Probiotic supplementation has been shown to benefit glycemic control, but existing evidence on β-cell function remains inconclusive. This meta-analysis investigated the effects of probiotic supplementation on pancreatic islet β-cell function, as assessed by the homeostasis model assessment for β-cell function (HOMA-β), in individuals with impaired glucose metabolism.

**Methods:**

A systematic search of PubMed, Embase, Web of Science, and the Cochrane Library was conducted. We included randomized controlled trials (RCTs) comparing probiotics with placebo or no additional treatment in adults with abnormal glucose metabolism. Random-effects meta-analysis was performed, accounting for the potential influence of heterogeneity.

**Results:**

Twelve RCTs involving 907 participants were included. Compared with controls, probiotic supplementation significantly improved HOMA-β (mean difference [MD]: 3.04, 95% CI: 0.23 to 5.86; *p* = 0.03; I^2^ = 92%). However, the sensitivity analysis limited to studies with low risk of bias did not show that probiotics have a significant influence on HOMA-β in these participants (MD: -1.31, 95% CI: −6.30 to 3.68, *p* = 0.61; I^2^ = 63%). Subgroup analysis showed a significant benefit in participants with baseline HbA1c ≥ 8.5% (MD: 7.05, 95% CI: 5.85 to 8.24; I^2^ = 0%), but not in those with HbA1c < 8.5% (MD: 0.19, 95% CI: −1.09 to 1.46; I^2^ = 37%; *p* for subgroup difference < 0.001). Meta-regression further confirmed that higher baseline HbA1c was positively associated with greater HOMA-β improvement (coefficient = 2.91; *p* = 0.04; adjusted R^2^ = 62.5%). Other factors, such as mean age, sex, baseline body mass index, HOMA-β, concurrent hypoglycemic treatment, or probiotic treatment duration, did not significantly affect the results.

**Conclusion:**

Probiotics might enhance islet β-cell function in individuals with glucose metabolism disorders, particularly among those with elevated baseline HbA1c levels. However, large-scale high-quality RCTs are needed to validate our findings.

**Systematic review registration:**

The protocol of the meta-analysis was registered at PROSPERO with the identifier CRD420251087101.

## Introduction

Glucose metabolism disorders encompass a spectrum of conditions characterized by hyperglycemia due to impaired insulin secretion, insulin resistance, or both (1). These include type 1 diabetes mellitus (T1DM), an autoimmune disorder leading to absolute insulin deficiency (2); type 2 diabetes mellitus (T2DM), characterized by insulin resistance with progressive β-cell failure (3); gestational diabetes mellitus (GDM), a transient form of glucose intolerance during pregnancy (4); and prediabetes, an intermediate state between normoglycemia and diabetes associated with increased cardiometabolic risk (5). Across these disorders, chronic hyperglycemia contributes to endothelial dysfunction, inflammation, and oxidative stress, leading to an increased risk of cardiovascular events and microvascular complications such as retinopathy, nephropathy, and neuropathy (6, 7). A key pathological feature underlying these conditions is the progressive failure of pancreatic β-cells, which leads to insufficient insulin secretion relative to metabolic demands (8).

Probiotics are live microorganisms that, when consumed in adequate amounts, confer health benefits on the host (9). Through modulation of the gut microbiota, enhancement of intestinal barrier function, and attenuation of systemic inflammation, probiotics are increasingly recognized for their potential metabolic effects (10, 11). Accumulating evidence suggests that probiotic supplementation may improve glycemic control, insulin sensitivity, and lipid metabolism in individuals with various glucose metabolism disorders, including diabetes mellitus (DM) (12, 13), prediabetes (14), and GDM (15). Proposed mechanisms include the production of short-chain fatty acids, regulation of incretin hormones such as glucagon-like peptide-1 (GLP-1), and reduction of endotoxemia and oxidative stress (16, 17), all of which may indirectly support β-cell preservation and function. However, despite the growing interest in this area, the specific impact of probiotics on islet β-cell function remains under-investigated (18). The homeostasis model assessment of β-cell function (HOMA-β), derived from fasting insulin and glucose levels, serves as a non-invasive and practical tool to estimate insulin secretory capacity (19). While several clinical trials have assessed the effect of probiotic supplementation on HOMA-β, results have been inconsistent and lack a comprehensive synthesis (20–31). Therefore, the aim of this meta-analysis was to systematically evaluate the effect of probiotics on islet β-cell function, measured by HOMA-β, in individuals with glucose metabolism disorders.

## Materials and methods

The guidelines set forth by PRISMA (Preferred Reporting Items for Systematic Reviews and Meta-Analyses) (32, 33) and the Cochrane Handbook (34) were followed in the design and performance of the study. The protocol of the meta-analysis was registered at PROSPERO with the identifier CRD420251087101.

### Study inclusion and exclusion criteria

The PICOS (Population, Intervention, Comparison, Outcome, Study design) principle was followed in designating the study inclusion criteria.

P (Population): The population of interest for this meta-analysis comprised adults (≥ 18 years) with glucose metabolism disorders, operationally defined in this study as clinical conditions characterized by chronic hyperglycemia due to impaired insulin secretion, insulin resistance, or both. This definition encompasses T1DM, T2DM, GDM, and prediabetes. Eligible participants were those diagnosed with these conditions according to the diagnostic criteria reported in the original studies. In general, T1DM was defined by autoimmune-mediated β-cell destruction and absolute insulin dependence, most often diagnosed based on clinical presentation and requirement of insulin therapy (35). T2DM was typically diagnosed using internationally recognized thresholds, such as fasting plasma glucose ≥ 7.0 mmol/L, 2-h plasma glucose ≥ 11.1 mmol/L on an oral glucose tolerance test (OGTT), or HbA1c ≥ 6.5% (35). GDM was defined as glucose intolerance first identified during pregnancy, using criteria such as the International Association of Diabetes and Pregnancy Study Groups (IADPSG) or local diagnostic guidelines (36). Prediabetes was generally diagnosed by impaired fasting glucose (fasting plasma glucose 5.6–6.9 mmol/L), impaired glucose tolerance (2-h plasma glucose 7.8–11.0 mmol/L during OGTT), or HbA1c between 5.7 and 6.4% (37). Individuals with normoglycemia were not included, as their HOMA-β values are generally preserved and outside the scope of our research question.

I (Exposure): Probiotic supplementation in addition to standard treatment, with no limitations to the type, composition, or dose of probiotics.
C (Comparison): Placebo or no additional treatment on the basis of standard treatment.
O (Outcome): Between-group differences of the changes of HOMA-β after treatment; HOMA-β was calculated as: 20 × fasting insulin (μU/mL) / [fasting plasma glucose (mmol/L) – 3.5].
S (Study Design): Parallel-group RCTs.

Reviews, editorials, preclinical studies, studies not designed as RCTs, crossover studies, studies involving patients with normoglycemia, not including probiotics as an intervention, comparing to active controls, or that did not report the outcome of HOMA-β were subsequently excluded from the meta-analysis. In cases where studies included overlapping patient populations, the study with the largest sample size was selected for inclusion in the meta-analysis.

### Database search

The PubMed, Embase, Cochrane Library, and Web of Science databases were searched using the combination of the following terms: (1) “probiotic” OR “prebiotic” OR “synbiotic” OR “probiotics” OR “prebiotics” OR “synbiotics” OR “lactobacillus” OR “lactobacilli” OR “bifidobacteria” OR “bifidobacterium”; (2) “HOMA-β” OR “HOMA-beta” OR “β-cell function” OR “beta-cell function” OR “C-peptide” OR “homeostasis model assessment” OR “HOMA” OR “beta cell function” OR “β cell function”; and (3) “random” OR “randomly” OR “randomized” OR “control” OR “allocated” OR “placebo” OR “randomised.” Only studies that included human subjects and were published as full-length articles in peer-reviewed journals in English or Chinese were considered for inclusion. We intentionally included “prebiotics” and “synbiotics” in the search strategy to ensure comprehensive retrieval and avoid missing any studies where probiotics might have been evaluated alongside these terms. However, we strictly applied the inclusion criteria to retain only studies assessing probiotic interventions in the final analysis. To ensure comprehensive coverage, the reference lists of relevant reviews and primary studies were also examined. The final search of the databases was completed on 31 May 2025.

### Data collection and quality evaluation

Two reviewers independently performed the literature search, data extraction, and methodological quality evaluation. Any discrepancies were resolved through discussion with the corresponding author. Duplicate records were first removed using EndNote software. Two reviewers independently screened titles and abstracts for relevance, and full texts of potentially eligible articles were then assessed in detail. Discrepancies at any stage were resolved by discussion with a third reviewer to ensure consistent application of inclusion and exclusion criteria. Extracted data included general study characteristics (e.g., first author, year of publication, and study location), design type (double-blind, single-blind, or open-label), participant demographics (including diagnosis, sample size, average age, sex distribution, and baseline values for body mass index (BMI), HbA1c, and HOMA-β), details regarding probiotic and control interventions, co-administered treatments, and duration of therapy. The methodological quality of RCTs included in this meta-analysis was assessed using the Cochrane Risk of Bias 2.0 (RoB 2) tool (34). This tool evaluates five domains of potential bias: bias arising from the randomization process, bias due to deviations from intended interventions, bias due to missing outcome data, bias in measurement of the outcome, and bias in selection of the reported result. Each domain was judged as having low risk of bias, some concerns, or high risk of bias, and an overall risk of bias judgment was assigned accordingly. The certainty of evidence was independently evaluated by two reviewers using the GRADE (Grading of Recommendations, Assessment, Development, and Evaluation) approach, which considers factors such as risk of bias, inconsistency, indirectness, imprecision, and potential publication bias (38). Evidence quality was rated as high, moderate, low, or very low. Any disagreements were resolved through consultation with the corresponding author.

### Statistical analysis

The influence of probiotics on HOMA-β of participants with glucose metabolism disorders, in comparison with controls, was summarized as mean difference (MD) and corresponding 95% confidence interval (CI) (34). Heterogeneity was assessed using the Cochrane Q test (34). The I^2^ statistic was also calculated, with I^2^ < 25%, 25–75%, and > 75% indicating mild, moderate, and substantial heterogeneity, respectively (39). A random-effects model based on the DerSimonian–Laird method was applied to synthesize the data, as it accounts for potential variability across studies (34). A sensitivity analysis limited to low-risk of bias RCTs was performed. Subgroup analysis was also conducted to evaluate the impact of study characteristics on outcomes, such as mean ages of the subjects, sex distribution, baseline BMI, HbA1c, HOMA-β, with or without concurrent hypoglycemic treatment, and treatment durations. For continuous variables, median values of study-level means were used as cutoffs to ensure a balanced number of studies in each subgroup, thereby improving the statistical power for testing between-subgroup differences. However, all subgroup analyses were based on study-level rather than individual-level data, so individual-level associations cannot be inferred. Moreover, univariate meta-regression analysis was also performed to investigate if the study characteristics in continuous variables could significantly modify the meta-analysis results (34). Publication bias was assessed through visual examination of funnel plots and Egger’s regression asymmetry test (40), with a *p*-value < 0.05 indicating statistical significance. All statistical analyses were performed using RevMan (version 5.3; Cochrane Collaboration, Oxford, United Kingdom) and Stata (version 17.0; StataCorp, College Station, TX, United States).

## Results

### Literature search

**Figure 1:**
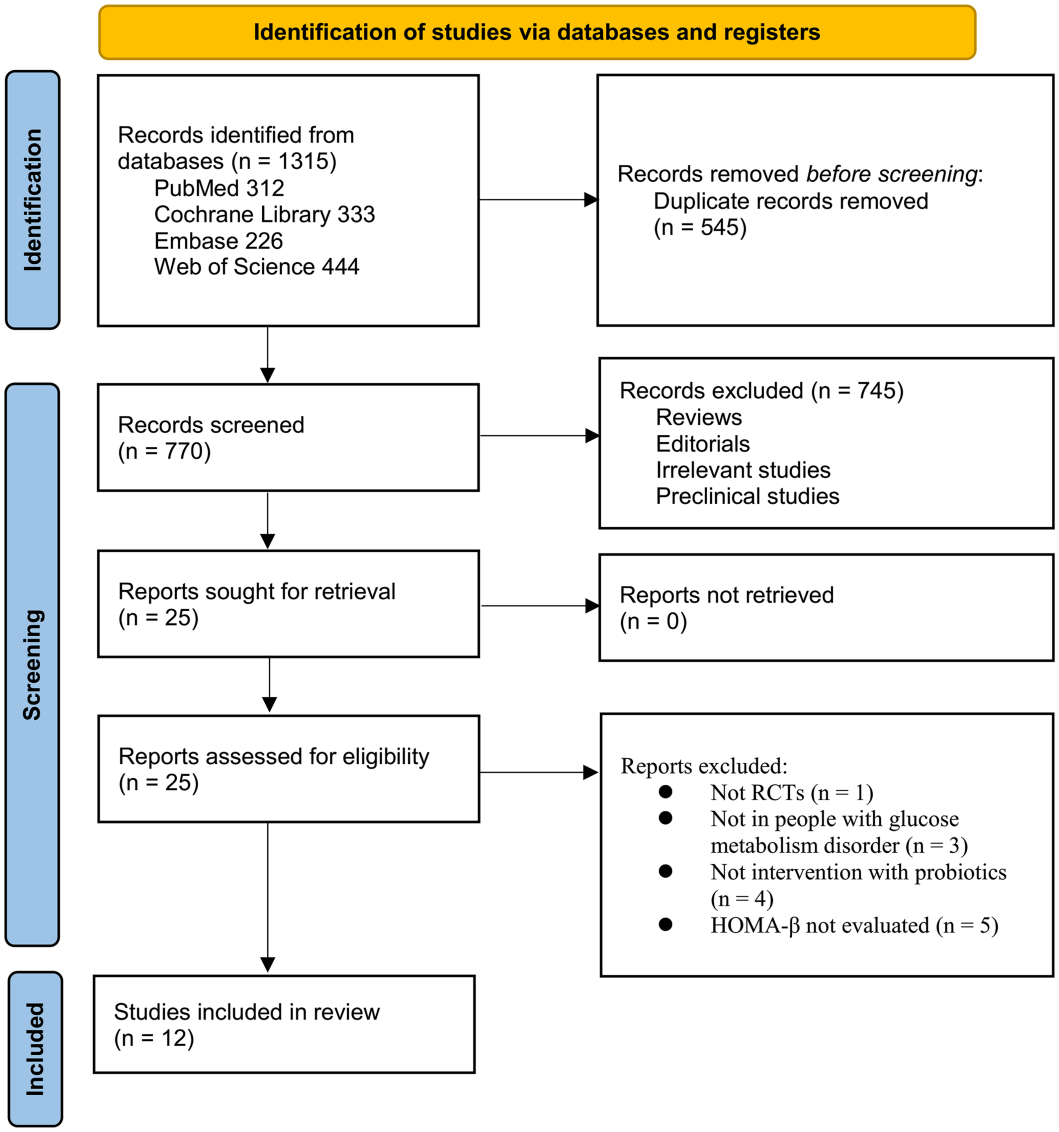
Flowchart for the literature search and study inclusion.

Figure 1 illustrates the study selection process. The initial database search yielded 1,315 records, which were reduced to 770 after removing duplicates. Title and abstract screening excluded 745 records due to irrelevance to the meta-analysis objective. The full texts of the remaining 25 articles were reviewed, resulting in the exclusion of 13 studies for reasons specified in Figure 1. Consequently, 12 RCTs (20–31) were included in the quantitative synthesis.

### Study characteristics

An overview of the included studies is provided in Table 1. This meta-analysis included 12 RCTs published between 2014 and 2025, conducted in Iran, China, and Ukraine. The total sample size was 907 participants with various glucose metabolism disorders: eight studies focused on patients with type 2 diabetes mellitus (T2DM) (20, 23, 25–31), one on gestational diabetes mellitus (GDM) (21), one on prediabetes (24), and another on T1/T2DM patients receiving hemodialysis (22). The mean age of participants ranged from 30.8 to 68.9 years, and the proportion of male patients ranged from 0 to 66.7%. Baseline body mass index (BMI) ranged from 22.0 to 32.6 kg/m^2^, and baseline HbA1c levels varied from 5.7 to 10.4%. The interventions consisted of single or multi-strain probiotic or synbiotic formulations—commonly including *Lactobacillus, Bifidobacterium,* and *Enterococcus* species—with doses ranging from 1 × 10^8^ to 6 × 10^10^ CFU per day. Control groups received a placebo in seven studies (20–22, 24, 28, 30, 31) or non-probiotic alternatives (23, 25–27, 29) in five studies. Concurrent antidiabetic treatments, including metformin, sulfonylureas, insulin, acarbose, sitagliptin, and dulaglutide, were also used in 10 studies (20, 22, 23, 25–31). Treatment durations ranged from 6 to 24 weeks.

**Table 1:** Characteristics of the included studies.

Study	Country	Design	Diagnosis	No. of patients	Mean age (years)	Men (%)	Baseline mean BMI (kg/m^2^)	Baseline mean HbA1c (%)	Baseline mean HOMA-β	Details of intervention (Probiotics)	Details of control	Concurrent treatment	Treatment duration (weeks)
Tajadadi 2014 (20)	Iran	R, DB, PC	T2DM	54	52.7	18.5	30.2	NR	18.3	*L. sporogenes* (1 * 10^8^ CFU/g) containing bread	Bread with no probiotics	Metformin and glibenclamide	8
Karamali 2016 (21)	Iran	R, DB, PC	GDM	60	30.8	0	28.6	NR	45	*Lactobacillus acidophilus, L. casei*, *Bifidobacterium bifidum* (2 * 10^9^ CFU/g each)	Placebo capsules (cellulose)	None	6
Xie 2017 (23)	China	R, OL	Newly diagnosed T2DM	128	54.3	59.4	24.5	10.4	39.2	Bifid-Triple Viable capsules (420 mg TID containing *Bifidobacterium, Lactobacillus acidophilus*, and *Enterococcus faecalis*)	No probiotics	Acarbose + insulin glargine	12
Soleimani 2017 (22)	Iran	R, DB, PC	T1/T2DM patients on hemodialysis	60	56.7	66.7	26.3	6	60.9	*Lactobacillus acidophilus, L. casei*, and *Bifidobacterium bifidum* (2 * 10^9^ CFU/g each strain)	Placebo capsule	Insulin	12
Kassaian 2018 (24)	Iran	R, DB, PC	Prediabetes	55	52.9	45.5	30	5.7	125.5	*Lactobacillus acidophilus, Bifidobacterium lactis, B. bifidum, B. longum* (1 * 10^9^ CFU each)	Placebo capsule	None	24
Zhang 2018 (25)	China	R, OL	T2DM with poor glycemic control	94	56.3	55.3	24.5	10.4	37.3	*Bifidobacterium* tetravaccine tablets (containing *Bifidobacterium, Lactobacillus acidophilus*, *Enterococcus faecalis*, and *Bacillus cereus*), 1.5 g tid	No probiotics	Oral hypoglycemic agents or insulin	10
Wu 2020 (26)	China	R, OL	Newly diagnosed T2DM	108	59	55.6	24.7	8.7	37.1	*Bifidobacterium* tetravaccine tablets, 1.5 g tid	No probiotics	Acarbose + insulin glargine	12
Hasgova 2021 (27)	China	R	Elderly T2DM	118	68.9	51.7	22.4	8.8	31.2	Triple viable *bifidobacterium*: *Bifidobacterium, Lactobacillus*, Enterococcus, 630 mg bid	No probiotics	Metformin + sitagliptin	12
Savytska 2023 (28)	Ukraine	R, DB, PC	T2DM	68	55.4	NR	32.2	8.9	33.5	Symbiter (14-strain probiotic: *Lactobacillus*, *Bifidobacterium, Propionibacterium*, Acetobacter; 6 * 10^10^ CFU/g), 10 g qd	Placebo capsule	Stable doses of metformin and/or sulfonylureas/insulin	8
Li 2024 (29)	China	R, OL	Newly diagnosed T2DM	56	55.3	57.1	22	8.4	21.1	*Bifidobacterium* quadruple viable bacteria (1.5 g tablets tid)	No probiotics	Metformin	12
Savytska 2024 (30)	Ukraine	R, DB, PC	T2DM	46	54.8	NR	32.6	8.4	43.6	Live multistrain probiotic (17 strains, including *Lactobacillus, Bifidobacterium, Lactococcus, Propionibacterium*, Acetobacter) 10 g/d	Placebo	Insulin therapy alone or combined with oral anti-diabetic drugs (metformin and/or sulfonylureas)	8
Tian 2025 (31)	China	R, DB, PC	T2DM	60	49.8	58.3	25.9	8.1	1.2	Probiotic capsules containing *Bifidobacterium longum*, *Lactobacillus acidophilus*, and *Enterococcus faecalis*, 2 * 10^9^ CFU/dose	Placebo	Dulaglutide and metformin	12

NR, not reported; R, randomized; DB, double-blind; PC, placebo-controlled; OL, open-label; T1DM, type 1 diabetes mellitus; T2DM, type 2 diabetes mellitus; GDM, gestational diabetes mellitus; CFU, colony-forming units; tid, three times daily; bid, twice daily; qd, once daily; BMI, body mass index; HbA1c, glycated hemoglobin; HOMA-β, homeostasis model assessment of β-cell function.

### Risk of bias evaluation

As summarized in Table 2, six studies were judged to be at low risk of bias across all domains using the Cochrane RoB 2 Tool (20–22, 28, 30, 31). Four studies (23, 25, 26, 29) were rated as high risk due to open-label designs and unclear randomization methods, and two (24, 27) were judged to have some concerns due to issues such as per-protocol analyses, lack of allocation concealment, or absence of a pre-registered protocol. Most studies used standardized and blinded laboratory methods to assess HOMA-β, minimizing measurement bias, and no major concerns were identified regarding selective reporting.

**Table 2:** Risk of bias evaluation via Cochrane Risk of Bias Tool 2.0 with reasons.

RCTs	Randomization process	Deviations from intended interventions	Missing outcome data	Measurement of the outcome	Selection of the reported results	Overall
Tajadadi 2014 (20)	Low risk (Computer-generated randomization, stratified by age/sex/BMI/medications. Allocation concealment maintained)	Low risk (Blinding of participants/personnel ensured, 5/81 dropped out. ITT analysis performed)	Low risk (ITT analysis with last-observation-carried-forward; attrition balanced across groups)	Low risk (Lab measurements blinded, duplicate assays, and standardized protocols)	Low risk (Pre-specified outcomes reported; no evidence of selective reporting)	Low risk
Karamali 2016 (21)	Low risk (Computer-generated randomization, stratified by age/BMI. Allocation concealment maintained)	Low risk (Blinding ensured, but 3/60 dropped out. ITT analysis performed)	Low risk (ITT analysis with last-observation-carried-forward; attrition <10%)	Low risk (Standardized lab assays with blinded duplicates; CV < 5%)	Low risk (Pre-specified outcomes reported; no selective reporting)	Low risk
Xie 2017 (23)	Some concerns (Randomization mentioned, but method not fully described; baseline characteristics balanced)	High risk (open-label design, no blinding of participants/personnel)	Low risk (No attrition reported; ITT analysis implied)	Low risk (Standardized lab assays; objective outcomes)	Low risk (Pre-specified outcomes reported; no evidence of selective reporting)	High risk (Due to open-label design)
Soleimani 2017 (22)	Low risk (Computer-generated random numbers were used, and allocation was concealed until analysis. Groups were well-matched at baseline)	Low risk (Double-blinding was maintained, and compliance was high: >90% capsule consumption. No significant deviations reported)	Low risk (5 participants withdrew, but ITT analysis was performed)	Low risk (Standardized laboratory methods)	Low risk (Pre-specified outcomes in the clinical trial registry were reported)	Low risk
Kassaian 2018 (24)	Low risk (Computer-generated random numbers with blocking stratified by age/gender. Allocation concealed until analysis)	Low risk (Double-blinding maintained; compliance >90%. Non-compliance led to exclusion)	Some concerns (Per-protocol analysis, excluded 35/120 participants, attrition due to antibiotics, and low compliance)	Low risk (Standardized laboratory methods)	Low risk (Pre-specified outcomes in the clinical trial registry were reported)	Some concerns (Due to per-protocol analysis and attrition)
Zhang 2018 (25)	Some concerns (No details on sequence generation or allocation concealment)	High risk (open-label design, though outcomes are lab-measured)	Low risk (No dropouts reported; all 94 participants completed the study)	Low risk (Standardized laboratory methods)	Some concerns (No pre-registered protocol found; potential selective reporting bias)	High risk (Due to lack of blinding and unclear randomization)
Wu 2020 (26)	Some concerns (Random number table used, but no details on allocation concealment)	High risk (open-label design, though outcomes are lab-measured)	Low risk (No dropouts reported; all 108 participants completed the study)	Low risk (Standardized laboratory methods)	Some concerns (No pre-registered protocol found; potential selective reporting bias)	High risk (Due to lack of blinding and unclear randomization)
Hasgova 2021 (27)	Some concerns (Random number table used, but no details on allocation concealment)	Low risk (No significant deviations reported; both groups received intended treatments)	Low risk (No dropouts mentioned; complete data reported)	Low risk (Standardized laboratory methods)	Some concerns (No pre-registered protocol; outcomes align with methods, but selective reporting is possible)	Some concerns (Due to unclear randomization and potential reporting bias)
Savytska 2023 (28)	Low risk (Computer-generated block randomization, allocation concealment maintained)	Low risk (Double-blinding ensured; per-protocol analysis with high compliance >90%)	Low risk (ITT analysis performed; 5 dropouts due to COVID-19)	Low risk (Standardized laboratory methods)	Low risk (Pre-specified primary outcome reported)	Low risk
Li 2024 (29)	Some concerns (Simple randomization used, but no details on allocation concealment)	High risk (open-label design; no placebo; performance bias likely)	Low risk (No dropouts reported; complete data for all 84 participants)	Low risk (Standardized laboratory methods)	Some concerns (No pre-registered protocol; outcomes align with methods, but selective reporting is possible)	High risk (Due to lack of blinding and unclear randomization)
Savytska 2024 (30)	Low risk (Randomization was double-blind, computer-generated, and performed by a statistical expert using blocks of four. Allocation concealment was maintained)	Low risk (The study was double-blinded, and adherence was monitored, consumed >85% of sachets)	Low risk (All randomized patients completed the study, and no significant missing data were reported)	Low risk (Standardized laboratory methods)	Low risk (Pre-specified outcomes in the clinical trial registry were reported)	Low risk
Tian 2025 (31)	Low risk (Computer-generated randomization with allocation concealment; baseline characteristics were balanced)	Low risk (Double-blinded design with identical placebo; adherence was high, ≥90%)	Low risk (No dropouts reported; all 60 participants completed the study)	Low risk (Standardized laboratory methods)	Low risk (Pre-specified outcomes in the clinical trial registry were reported)	Low risk

RCTs, randomized controlled trials; ITT, intention-to-treat; CV, coefficient of variation; T1DM, type 1 diabetes mellitus; T2DM, type 2 diabetes mellitus; DB, double-blind; PC, placebo-controlled.

### Influence of probiotics on HOMA-β

**Figure 2:**
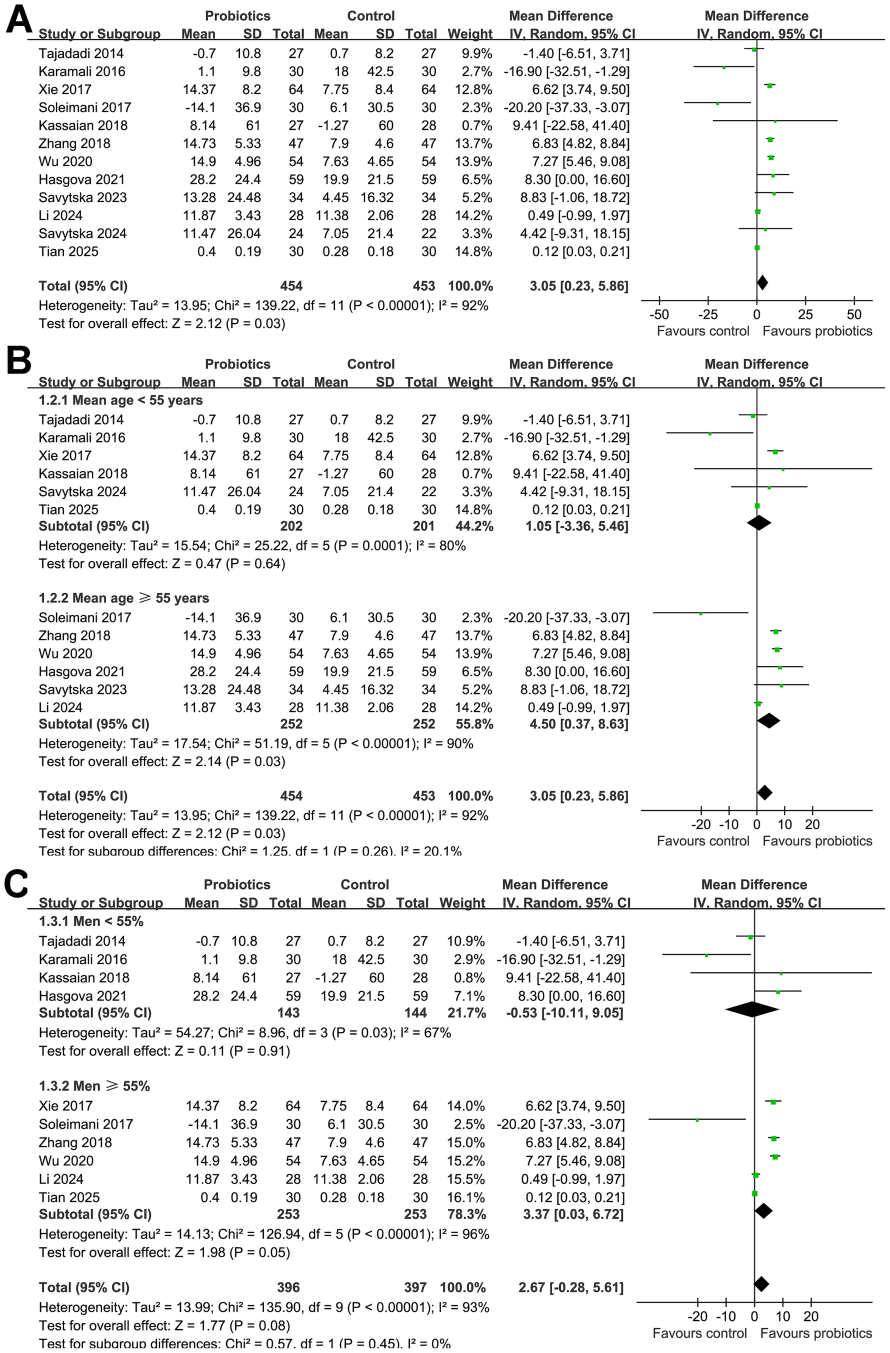
Forest plots for the meta-analysis evaluating the influence of probiotics on HOMA-β in participants with glucose metabolism disorders. **(A)** Overall meta-analysis; **(B)** subgroup analysis according to the mean ages of the subjects; and **(C)** subgroup analysis according to the proportions of men.

Overall, 454 subjects were allocated to the intervention group of probiotics, and 453 participants received controls. The pooled results of the 12 RCTs (20–31) showed that compared to controls, supplementation with probiotics significantly improved HOMA-β in participants with glucose metabolism disorders (MD: 3.04, 95% CI: 0.23 to 5.86, *p* = 0.03; Figure 2A) with considerable heterogeneity (*p* for Cochrane Q test < 0.001; I^2^ = 92%). However, the sensitivity analysis is limited to studies with a low risk of bias (20–22, 28, 30, 31) did not show that probiotics have a significant influence on HOMA-β in these participants (MD: −1.31, 95% CI: −6.30 to 3.68, *p* = 0.61; I^2^ = 63%; Supplementary Figure 1).

**Figure 3:**
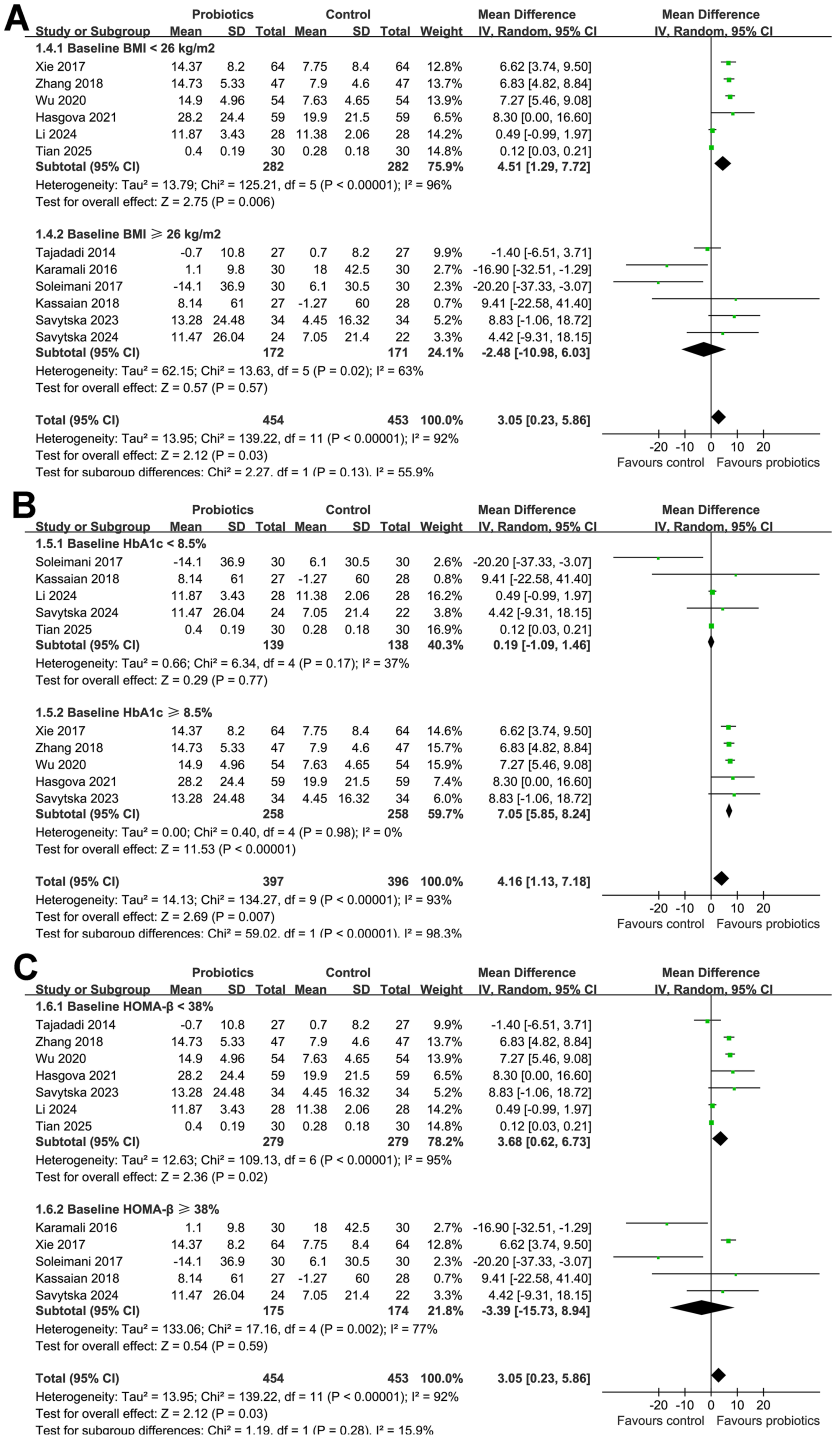
Forest plots for the subgroup analyses evaluating the influence of probiotics on HOMA-β in participants with glucose metabolism disorders. **(A)** Subgroup analysis according to baseline mean BMI; **(B)** subgroup analysis according to baseline mean HbA1c; and **(C)** subgroup analysis according to baseline mean HOMA-β.

**Figure 4:**
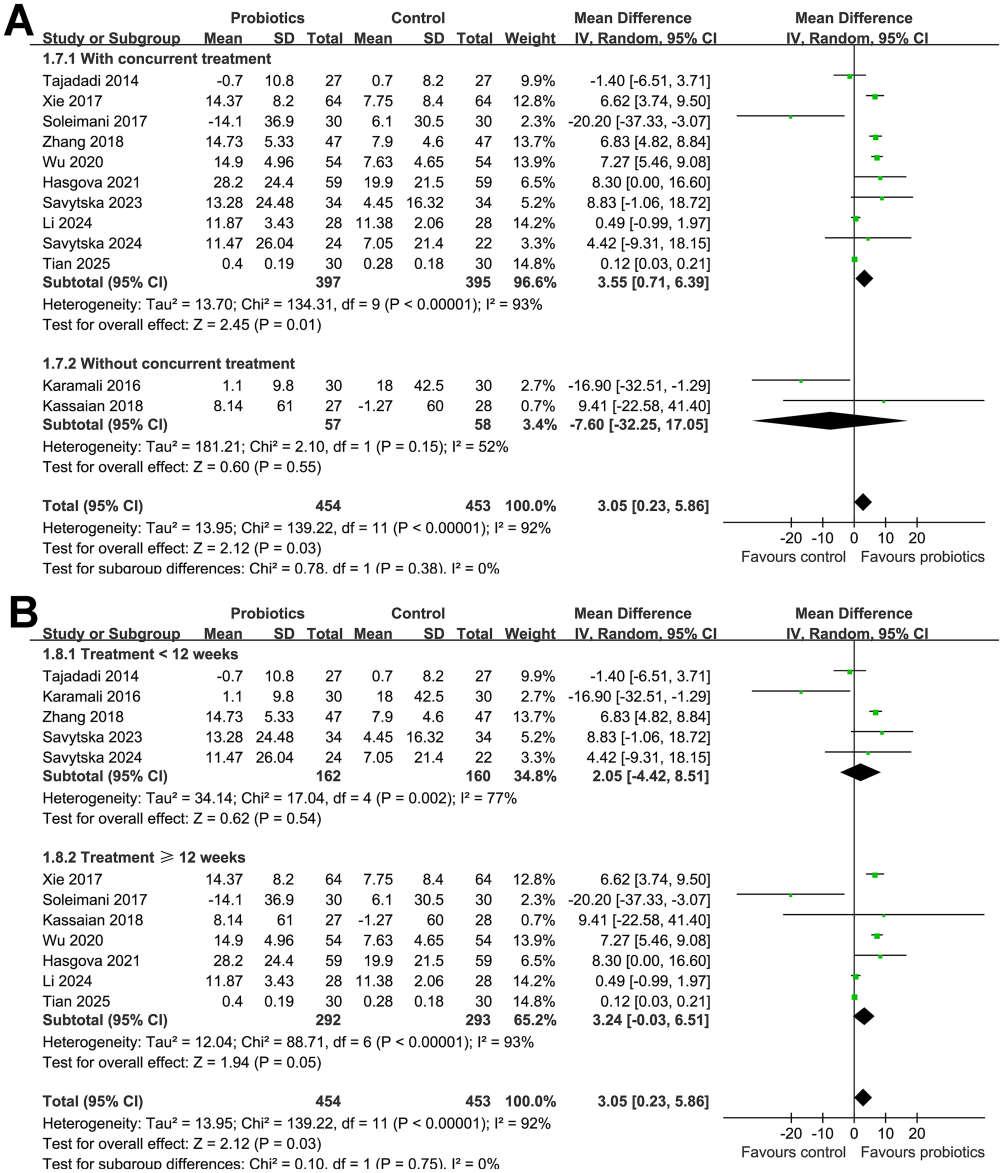
Forest plots for the subgroup analyses evaluating the influence of probiotics on HOMA-β in participants with glucose metabolism disorders. **(A)** Subgroup analysis between studies with and without concurrent hypoglycemic treatments; and **(B)** subgroup analysis according to the treatment duration of probiotics.

Subsequent subgroup analyses did not show that the results were significantly affected by the mean age of the participants (*p* for subgroup difference = 0.26; Figure 2B), proportion of men (*p* for subgroup difference = 0.45; Figure 2C), or mean BMI of the subjects at baseline (*p* for subgroup difference = 0.13; Figure 3A). Interestingly, we found that probiotics significantly improved HOMA*-*β in subjects with baseline HbA1c ≥ 8.5% (MD: 7.05, 95% CI: 5.85 to 8.24, *p* < 0.001; I^2^ = 0%), but not in subjects with baseline HbA1c < 8.5% (MD: 0.19, 95% CI: −1.09 to 1.46, *p* < 0.001; I^2^ = 37%; *p* for subgroup difference <0.001; Figure 3B). Further subgroup analyses did not suggest that the effect of probiotics on HOMA*-*β could be significantly affected by baseline HOMA*-*β (*p* for subgroup difference = 0.28; Figure 3C), with and without concurrent hypoglycemic treatments (*p* for subgroup difference = 0.38; Figure 4A), or treatment durations of probiotics (*p* for subgroup difference = 0.75; Figure 4B).

**Figure 5:**
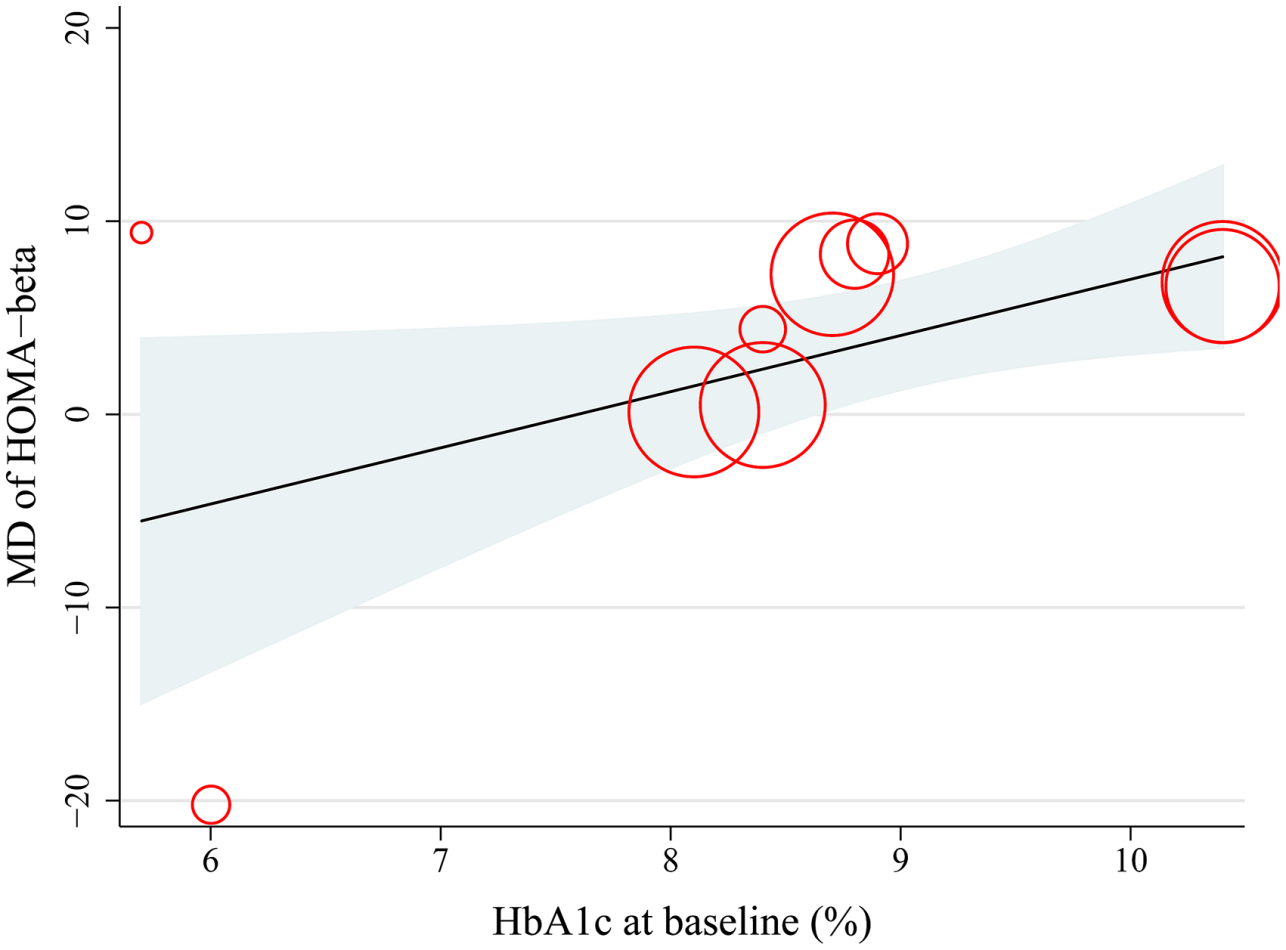
Plots for the meta-regression analysis evaluating the correlation of HbA1c at baseline with the improvement of HOMA-β after probiotics supplementation.

Finally, results of the univariate meta-regression analysis showed that a higher baseline HbA1c was significantly associated with greater improvement in HOMA-β (coefficient = 2.91, *p* = 0.04; Figure 5; Table 3), which largely explained the source of heterogeneity (Adjusted R^2^ = 62.5%). Other variables, such as mean age, proportion of men, baseline BMI, baseline HOMA*-*β, or treatment duration, did not seem to significantly modify the effect of probiotics on HOMA-β (*p* all >0.05; Table 3).

**Table 3:** Results of univariate meta-regression analysis for the influence of probiotics on HOMA-β in patients with glucose metabolism disorders.

Variables	MD in changes of HOMA-β between probiotics and control groups after treatment			
	Coefficient	95% CI	*p* value	Adjusted R^2^
Mean age (years)	0.65	−0.09 to 1.39	0.10	7.5%
Men (%)	0.18	−0.13 to 0.49	0.22	24.8%
Baseline BMI (kg/m^2^)	−0.31	−1.76 to 1.13	0.64	0%
Baseline HbA1c (%)	2.91	0.19 to 5.62	0.04	62.5%
Baseline HOMA-β	0.034	−0.233 to 0.302	0.78	0%
Treatment duration (weeks)	0.74	−1.26 to 2.73	0.43	0%

MD, mean difference; CI, confidence interval; BMI, body mass index; HbA1c, glycated hemoglobin; HOMA-β, homeostasis model assessment of β-cell function.

### Publication bias

**Figure 6:**
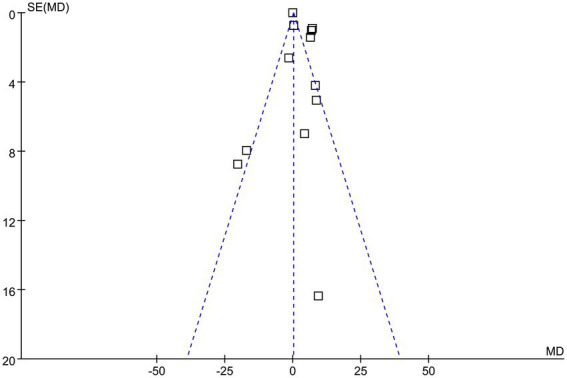
Funnel plots evaluating the publication bias underlying the meta-analysis evaluating the influence of probiotics on HOMA-β in participants with glucose metabolism disorders.

The funnel plots for the meta-analysis evaluating the effect of probiotics on HOMA-β in participants with glucose metabolism disorders are shown in Figure 6. These plots are symmetrical on visual inspection, suggesting a low risk of publication bias. Egger’s regression test also indicated a low risk of publication bias (*p* = 0.32).

### Certainty of evidence

The summarized certainty of evidence table using the GRADE system is shown in Table 4. The certainty of evidence was downgraded by one level to moderate because some of the included studies had high or unclear risk in the method of randomization and blinding.

**Table 4:** Summarized certainty of evidence using the GRADE system.

Outcome	No. of participants (studies)	Design	Risk of bias	Inconsistency	Indirectness	Imprecision	Other considerations	Relative effect (95% CI)	Certainty of evidence (GRADE)	Comments
Change of HOMA-β (probiotics vs. placebo or no additional treatment)	907 (12 RCTs)	Randomized controlled trials	Serious Some studies had a high or unclear risk in the method of randomization and blinding	Not serious Substantial heterogeneity (I^2^ = 92%), but the source of heterogeneity was explained in subgroup and meta-regression analyses	Not serious Population, interventions, and outcomes are directly relevant	Not serious 95% CI excludes no effect and is clinically meaningful	None	MD: 3.05% (95% CI: 0.23–5.86)	⨁⨁⨁◯ Moderate	Probiotics improve HOMA-β compared with controls in subjects with glucose metabolism disorders. Downgraded for study-level randomization or blinding limitations.

GRADE, Grading of Recommendations, Assessment, Development and Evaluation; RCTs, randomized controlled trials; MD, mean difference; CI, confidence interval; HOMA-β, homeostasis model assessment of β-cell function. Specific reasons for each GRADE domain, including: Risk of bias: Downgraded if a significant proportion of studies had unclear or high risk of bias in key domains (e.g., random sequence generation, allocation concealment, or selective reporting). Inconsistency: Downgraded if substantial heterogeneity was observed (I^2^ > 50%) and could not be explained by subgroup analyses or meta-regression. Indirectness: Evaluated but not downgraded, as all included studies directly assessed the population and outcomes of interest. Imprecision: Downgraded if confidence intervals were wide, overlapping, no effect, or if the overall sample size was small. Publication bias: Assessed using funnel plots and Egger’s test; downgraded if significant asymmetry suggested potential bias.

## Discussion

This meta-analysis synthesized current evidence from 12 RCTs and demonstrated that probiotic supplementation significantly improves islet β-cell function, as reflected by HOMA-β, in individuals with glucose metabolism disorders. While considerable heterogeneity was observed across the included studies (I^2^ = 92%), this represents an important limitation of our analysis. The sensitivity analysis restricted to high-quality RCTs did not show a significant effect of probiotics on HOMA-β, suggesting that study quality may partly explain the inconsistent findings. Therefore, the pooled results should be interpreted with caution and considered exploratory rather than definitive. Our subgroup and meta-regression analyses indicated that baseline HbA1c was a major effect modifier, explaining more than 60% of the heterogeneity, which suggests that differences in baseline glycemic control are likely a key driver of variability across studies. Nevertheless, residual heterogeneity remained, and the possibility that the overall positive effect was influenced by lower-quality trials cannot be excluded. These observations highlight the need for future large-scale, rigorously designed RCTs with standardized probiotic formulations and longer follow-up to more definitively determine the impact of probiotics on β-cell function. While the overall mean difference in HOMA-β was statistically significant, its clinical relevance remains uncertain. There is currently no universally accepted minimal clinically important difference (MCID) for HOMA-β, and prior studies rarely define thresholds for meaningful improvement. The lower bound of the 95% CI (0.23%) suggests that, for some patients, the absolute change may be too small to produce measurable clinical benefit. Therefore, the observed improvement should be interpreted as hypothesis-generating rather than conclusive for clinical decision-making.

Compared with recent meta-analyses focusing on probiotics in specific populations or general glycemic outcomes (41, 42), our study provides a broader, cross-disorder analysis specifically targeting β-cell function, incorporating data across T1DM, T2DM, GDM, and prediabetes. The identification of baseline HbA1c ≥ 8.5% as a key effect modifier, explaining 62.5% of the heterogeneity, suggests that probiotic benefits may be most pronounced in patients with poor glycemic control, offering clinically relevant insights for personalized therapy. Although the overall effect size (MD: 3.04%) was smaller than in some prior reports, this likely reflects differences in study populations, intervention heterogeneity, and analytic methods. By providing updated evidence through May 2025 and systematically exploring heterogeneity sources, our analysis refines the understanding of probiotics’ role in metabolic health while emphasizing the need for standardized interventions, longer-term RCTs, and mechanistic studies to translate these findings into clinical recommendations. This meta-analysis focused on HOMA-β because β-cell function has been rarely evaluated in previous probiotic meta-analyses, which predominantly examined glycemic control indices such as HbA1c or fasting glucose. Although single-sample HOMA modeling has greater intra-subject variability than multiple-sample approaches, it remains the most widely used and feasible method for β-cell function assessment in clinical trials, enabling standardized comparisons across studies. Consistent with our results, Singh et al. reported significant reductions in HbA1c, fasting glucose, and HOMA-IR in T2DM patients receiving probiotics, suggesting metabolic benefits beyond glycemic control alone (43). However, while Singh et al. focused on glycemic indices in T2DM, our analysis specifically evaluated β-cell function (HOMA-β) across diverse glucose metabolism disorders. The comparison highlights that although probiotics may improve glucose homeostasis in T2DM, their effects on β-cell function remain less established and may be more pronounced in patients with poor baseline glycemic control, as suggested by our subgroup analyses.

The beneficial effect of probiotics on HOMA-β can be attributed to several plausible physiological mechanisms. Probiotics have been shown to influence host metabolism through modulation of the gut microbiota, leading to increased production of short-chain fatty acids (SCFAs) such as butyrate, propionate, and acetate (44, 45). These metabolites improve insulin sensitivity, enhance GLP-1 secretion, and reduce systemic inflammation—all of which indirectly support β-cell function (46, 47). Furthermore, probiotics can strengthen intestinal barrier integrity, thereby reducing the translocation of endotoxins and lowering chronic low-grade inflammation (48, 49), which is known to impair β-cell survival and function (50). Some probiotic strains may also exert antioxidant effects or directly interact with enteroendocrine and immune cells, creating a more favorable metabolic and inflammatory environment for insulin secretion (51, 52). However, the core molecular pathways that mediate the benefit of probiotics on islet β-cell function remain to be determined in future studies. Importantly, no included RCT directly investigated the mechanistic effects of probiotics on β-cell function. Therefore, the proposed pathways, including modulation of gut microbiota, SCFA production, incretin regulation, and anti-inflammatory effects, remain largely speculative and are extrapolated from preclinical or indirect evidence. These mechanisms require confirmation in dedicated mechanistic studies before firm conclusions can be drawn.

An important and novel finding of this study lies in the differential impact of probiotics based on baseline HbA1c levels. Subgroup analysis revealed that probiotic supplementation significantly improved HOMA-β in individuals with a baseline HbA1c ≥ 8.5%, while no significant benefit was observed in those with a HbA1c < 8.5%. This finding was further supported by meta-regression analysis, which showed a positive association between baseline HbA1c and the magnitude of improvement in HOMA-β. One potential explanation for this finding is that individuals with poorly controlled glycemia experience greater gut dysbiosis, increased endotoxemia, and more pronounced β-cell stress, rendering them more responsive to the anti-inflammatory and metabolic effects of probiotics (53). In contrast, those with mild hyperglycemia may already have relatively preserved β-cell function and less microbial imbalance, limiting the potential for measurable improvement. These findings suggest that the metabolic benefits of probiotics may be most pronounced in patients with more severe glucose dysregulation, supporting the rationale for targeted supplementation strategies. Importantly, the significant improvement in HOMA-β was observed only in patients with HbA1c ≥ 8.5%, whereas those with HbA1c < 8.5% showed no benefit. This finding suggests that the overall pooled effect may be driven primarily by patients with poor baseline glycemic control, who likely have greater β-cell dysfunction and metabolic stress. Clinically, this indicates that probiotic supplementation, if beneficial, might be most appropriate for selected high-risk populations rather than applied broadly to all individuals with glucose metabolism disorders. Future trials should therefore stratify analyses by baseline glycemic status and explore whether targeted supplementation yields clinically meaningful metabolic improvements. In addition, because our meta-regression was performed using study-level rather than individual-level data, the results are subject to ecological fallacy, and individual-level associations cannot be directly inferred. Moreover, although baseline HbA1c explained 62.5% of the between-study heterogeneity, considerable residual variability remains, suggesting that additional unmeasured factors likely influence the effect of probiotics on β-cell function.

Notably, the sensitivity analysis, including only high-quality RCTs, showed no significant effect of probiotics on HOMA-β, and heterogeneity, although reduced, remained moderate (I^2^ = 63%). This finding underscores that methodological limitations and clinical diversity—such as probiotic strain composition, CFU dose, and treatment duration—likely contributed to the observed heterogeneity. Therefore, the pooled results should be considered exploratory rather than definitive, highlighting the need for larger, rigorously designed RCTs with standardized interventions to clarify the true effect of probiotics on β-cell function.

This meta-analysis has several notable strengths. First, the literature search was comprehensive and up to date, covering multiple major databases through May 2025 and including only RCTs, thereby enhancing the internal validity of the findings. Second, the analysis included a wide spectrum of glucose metabolism disorders (e.g., DM, GDM, and prediabetes), improving generalizability to various clinical contexts. Third, extensive subgroup and meta-regression analyses were conducted to explore the influence of study-level covariates, such as age, sex, BMI, baseline HbA1c, and HOMA-β, treatment duration, and concurrent hypoglycemic therapy. These exploratory analyses allowed for the identification of baseline HbA1c as a key determinant of probiotic efficacy, helping to clarify heterogeneity and support the robustness of the primary outcome.

Nonetheless, some limitations should be acknowledged. First, the follow-up durations of the included RCTs were relatively short (6–24 weeks), so the long-term sustainability and clinical significance of the observed improvements remain uncertain. Future large-scale trials with extended follow-up are warranted to determine whether probiotic supplementation yields durable metabolic benefits. On the other hand, although subgroup analysis by treatment duration showed no significant effect modification, this may be due to insufficient statistical power rather than the true absence of a duration effect. Future adequately powered RCTs with longer intervention periods are required to clarify whether treatment duration influences the metabolic benefits of probiotics. Second, we restricted the search to English and Chinese publications, which may introduce language bias and limit the generalizability of our findings, although these languages account for a substantial proportion of probiotic research output. The geographic concentration of included studies, primarily from Asia, and the restriction to English and Chinese publications, raise the possibility of publication and language bias. Moreover, not all included trials were placebo-controlled, and several used open-label designs, which introduce potential performance bias (23, 25, 26, 29). Although the outcome measure of HOMA-β is laboratory-based and less susceptible to subjective bias, lack of blinding may still influence study conduct and participant adherence. The overall number of included studies and sample size remained limited, which constrained the power of some subgroup comparisons and limited our ability to assess publication bias with a high degree of certainty, and selective reporting cannot be completely ruled out, especially in trials without pre-registered protocols. Another limitation of this study is the clinical heterogeneity of the included populations. Our analysis pooled participants with prediabetes, T2DM, GDM, and diabetes with complications, which represent distinct conditions with differing pathophysiology, clinical course, and treatment responses. The wide range of baseline HbA1c values (5.7–10.4%) and BMI (22.0–32.6 kg/m^2^) reflects this diversity and may have contributed to the heterogeneity of the results. We chose to pool these groups because the number of eligible RCTs reporting HOMA-β was limited, and all of these conditions share impaired β-cell function as a central feature. Nevertheless, this approach introduces methodological limitations, and the pooled estimates should be interpreted as exploratory. Our subgroup and meta-regression analyses suggest that baseline glycemic status, particularly HbA1c level, rather than diagnostic category per se, was the primary modifier of the observed probiotic effect. Future RCTs are needed to evaluate the impact of probiotics within specific, clinically homogeneous populations to provide more definitive conclusions. Additionally, the composition, strains, dosage, and duration of probiotic interventions varied widely across studies, which may have introduced clinical heterogeneity and influenced outcomes in ways not fully captured by the subgroup analyses. Specifically, the included studies used diverse probiotic formulations, ranging from single- to multi-strain products with different species and CFU counts. Such variability likely contributes to the clinical heterogeneity observed and limits the generalizability of our findings. Without standardized probiotic preparations, it remains difficult to provide specific clinical recommendations, underscoring the need for future trials with uniform interventions to clarify efficacy. Moreover, the inclusion of open-label studies with unclear randomization methods likely introduced performance and detection bias. Although HOMA-β is a laboratory-based outcome, lack of blinding may still affect study conduct, participant adherence, or laboratory handling. The sensitivity analysis excluding high-risk studies showed no significant effect, underscoring that the overall findings should be interpreted with caution. In addition, while HOMA-β is a widely accepted surrogate marker for β-cell function, it does not directly measure insulin secretion dynamics and may be affected by concurrent insulin resistance. Moreover, crossover studies were excluded to avoid carryover effects and enhance methodological consistency; no major crossover trials meeting eligibility criteria were identified that would likely alter our conclusions. Finally, it should be acknowledged that there is no universally accepted overarching definition or standardized index for “glucose metabolism disorders.” In this meta-analysis, we operationally defined this spectrum to include T1DM, T2DM, GDM, and prediabetes, based on diagnostic criteria reported in the original studies. Although this approach reflects their shared pathophysiological basis of hyperglycemia and β-cell dysfunction, the lack of a universal definition may introduce variability across studies and represents an inherent limitation of our work.

From a clinical perspective, the mean difference of 3.04% in HOMA-β represents a modest but statistically significant improvement in insulin secretory function. While the absolute clinical impact of this magnitude is difficult to interpret in isolation, the subgroup findings suggest that specific patient populations—particularly those with suboptimal glycemic control—may derive more substantial metabolic benefit. Given the safety profile and accessibility of probiotic supplementation, these results support its potential use as an adjunctive strategy for patients with glucose metabolism disorders, especially those with elevated HbA1c or early β-cell dysfunction (54). However, it remains unclear whether the observed improvements in HOMA-β translate into meaningful long-term outcomes, such as reduced need for pharmacotherapy, delayed disease progression, or fewer complications. Future research should aim to address several gaps. Large-scale, well-designed RCTs are needed to validate the effect of probiotics on β-cell function using more direct and dynamic measures, such as insulin secretion rates during oral glucose tolerance tests or mixed-meal tolerance tests, and the gold standard of the hyperglycemic clamp technique (55). Trials should aim for longer durations, standardized probiotic formulations, and rigorous blinding protocols. In addition, studies should assess whether improvements in HOMA-β are sustained over time and lead to clinical endpoints such as improved HbA1c control, reduced medication burden, or prevention of diabetes onset in high-risk individuals. Exploring microbiome profiles as predictive biomarkers of response may also help tailor probiotic therapy more precisely (56).

It should also be noted that the use of HOMA-β as the primary outcome carries important limitations. Single-sample HOMA-β has been reported to show higher intrasubject variability (coefficient of variation ≈7–8%) compared with repeated-sample approaches (≈4%), which may reduce measurement precision (57). Furthermore, HOMA-β does not always correlate linearly with insulin secretory capacity across different stages of glucose tolerance, and its interpretation can be complex in individuals with significant insulin resistance (58). Unlike dynamic tests such as the hyperglycemic clamp or oral glucose tolerance test, HOMA-β provides only an indirect, surrogate estimate of β-cell function. However, HOMA-β remains the most widely used and consistently reported measure of β-cell function in clinical trials, making it the only feasible parameter for meta-analysis across heterogeneous studies. We therefore emphasize that our results should be interpreted with caution and as hypothesis-generating, while future high-quality RCTs should incorporate more robust and standardized dynamic methods to better assess the impact of probiotics on β-cell function. Finally, it is important to recognize that, despite growing interest, the clinical evidence supporting probiotics for improving β-cell function in glucose metabolism disorders remains conflicting. No consensus statement or official guideline currently endorses the use of probiotics for this purpose. Our findings, therefore, should not be interpreted as a recommendation for clinical practice, but rather as hypothesis-generating evidence that may help guide future investigations. Large-scale, standardized, and longer-term RCTs will be essential to establish whether probiotic supplementation confers reproducible metabolic benefits and whether these findings can ultimately inform clinical guidelines.

## Conclusion

In summary, this meta-analysis suggests that probiotic supplementation may modestly improve islet β-cell function, as assessed by HOMA-β, in adults with glucose metabolism disorders. However, these findings should be interpreted with caution. Substantial statistical heterogeneity was present, and sensitivity analysis limited to high-quality studies did not confirm a significant benefit, raising the possibility that the pooled positive effect was influenced by lower-quality trials. In addition, the clinical diversity of the included populations—ranging from prediabetes to T2DM, GDM, and diabetes with complications—introduces methodological limitations, as these conditions differ in pathophysiology and treatment response. The reliance on HOMA-β as a surrogate outcome, which is subject to variability and interpretive complexity, further restricts the certainty of our conclusions.

At present, evidence regarding probiotic supplementation in this context remains conflicting, and no consensus statement or clinical guideline recommends probiotics to improve β-cell function in patients with glucose metabolism disorders. Therefore, our results should be regarded as exploratory and hypothesis-generating rather than conclusive. Future large-scale, rigorously designed RCTs are needed, incorporating standardized probiotic formulations, longer intervention and follow-up periods, and more robust dynamic assessments of β-cell function. Such studies will be essential to determine whether probiotics provide consistent clinical benefits and whether their use should ultimately be considered in clinical practice.

## Data Availability

The original contributions presented in the study are included in the article/Supplementary material, further inquiries can be directed to the corresponding author.

## References

[1] Perez JA. Glucose disorders. Prim Care. (2024) 51:375–90. doi: 10.1016/j.pop.2024.03.003, PMID: 39067965

[2] Haller MJ Bell KJ Besser REJ Casteels K Couper JJ Craig ME . ISPAD clinical practice consensus guidelines 2024: screening, staging, and strategies to preserve beta-cell function in children and adolescents with type 1 diabetes. Horm Res Paediatr. (2024) 97:529–45. doi: 10.1159/000543035, PMID: 39662065 PMC11854978

[3] Dludla PV Mabhida SE Ziqubu K Nkambule BB Mazibuko-Mbeje SE Hanser S . Pancreatic β-cell dysfunction in type 2 diabetes: implications of inflammation and oxidative stress. World J Diabetes. (2023) 14:130–46. doi: 10.4239/wjd.v14.i3.130, PMID: 37035220 PMC10075035

[4] Mittal R Prasad K Lemos JRN Arevalo G Hirani K. Unveiling gestational diabetes: an overview of pathophysiology and management. Int J Mol Sci. (2025) 26:2320. doi: 10.3390/ijms26052320, PMID: 40076938 PMC11900321

[5] Lizarzaburu-Robles JC Herman WH Garro-Mendiola A Galdón Sanz-Pastor A Lorenzo O. Prediabetes and Cardiometabolic risk: the need for improved diagnostic strategies and treatment to prevent diabetes and cardiovascular disease. Biomedicine. (2024) 12:363. doi: 10.3390/biomedicines12020363, PMID: 38397965 PMC10887025

[6] Bhatti JS Sehrawat A Mishra J Sidhu IS Navik U Khullar N . Oxidative stress in the pathophysiology of type 2 diabetes and related complications: current therapeutics strategies and future perspectives. Free Rad Biol Med. (2022) 184:114–34. doi: 10.1016/j.freeradbiomed.2022.03.019, PMID: 35398495

[7] Yang DR Wang MY Zhang CL Wang Y. Endothelial dysfunction in vascular complications of diabetes: a comprehensive review of mechanisms and implications. Front Endocrinol. (2024) 15:1359255. doi: 10.3389/fendo.2024.1359255, PMID: 38645427 PMC11026568

[8] Kerper N Ashe S Hebrok M. Pancreatic β-cell development and regeneration. Cold Spring Harb Perspect Biol. (2022) 14:741. doi: 10.1101/cshperspect.a040741 PMC9159263 34580120

[9] Hill C Guarner F Reid G Gibson GR Merenstein DJ Pot B . The international scientific Association for Probiotics and Prebiotics consensus statement on the scope and appropriate use of the term probiotic. Nat Rev Gastroenterol Hepatol. (2014) 11:506–14. doi: 10.1038/nrgastro.2014.66, PMID: 24912386

[10] Ma T Shen X Shi X Sakandar HA Quan K Li Y . Targeting gut microbiota and metabolism as the major probiotic mechanism—an evidence-based review. Trend Food Sci Tech. (2023) 138:178–98. doi: 10.1016/j.tifs.2023.06.013

[11] Chandrasekaran P Weiskirchen S Weiskirchen R. Effects of probiotics on gut microbiota: an overview. Int J Mol Sci. (2024) 25:6022. doi: 10.3390/ijms25116022, PMID: 38892208 PMC11172883

[12] Li G Feng H Mao XL Deng YJ Wang XB Zhang Q . The effects of probiotics supplementation on glycaemic control among adults with type 2 diabetes mellitus: a systematic review and meta-analysis of randomised clinical trials. J Transl Med. (2023) 21:442. doi: 10.1186/s12967-023-04306-0, PMID: 37415167 PMC10324246

[13] Moravejolahkami AR Shakibaei M Fairley AM Sharma M. Probiotics, prebiotics, and synbiotics in type 1 diabetes mellitus: a systematic review and meta-analysis of clinical trials. Diabetes Metab Res Rev. (2024) 40:e3655. doi: 10.1002/dmrr.3655, PMID: 37183580

[14] Li Y Wu Y Wu L Qin L Liu T. The effects of probiotic administration on patients with prediabetes: a meta-analysis and systematic review. J Transl Med. (2022) 20:498. doi: 10.1186/s12967-022-03695-y, PMID: 36324119 PMC9632036

[15] Mu J Guo X Zhou Y Cao G. The effects of probiotics/Synbiotics on glucose and lipid metabolism in women with gestational diabetes mellitus: a Meta-analysis of randomized controlled trials. Nutrients. (2023) 15:375. doi: 10.3390/nu15061375, PMID: 36986107 PMC10056932

[16] Zhai L Wu J Lam YY Kwan HY Bian ZX Wong HLX. Gut-microbial metabolites, probiotics and their roles in type 2 diabetes. Int J Mol Sci. (2021) 22:846. doi: 10.3390/ijms222312846, PMID: 34884651 PMC8658018

[17] Pintarič M Langerholc T. Probiotic mechanisms affecting glucose homeostasis: a scoping review. Life. (2022) 12:187. doi: 10.3390/life12081187, PMID: 36013366 PMC9409775

[18] Kobyliak N Khomenko M Falalyeyeva T Fedchenko A Savchuk O Tseyslyer Y . Probiotics for pancreatic β-cell function: from possible mechanism of action to assessment of effectiveness. Crit Rev Microbiol. (2024) 50:663–83. doi: 10.1080/1040841x.2023.2257776, PMID: 37705353

[19] Reaven GM. HOMA-beta in the UKPDS and ADOPT. Is the natural history of type 2 diabetes characterised by a progressive and inexorable loss of insulin secretory function? Maybe? Maybe not? Diab Vasc Dis Res. (2009) 6:133–8. doi: 10.1177/1479164109336038 20368203

[20] Tajadadi M Bahmani F Shakeri H Hadaegh H Hijijafari M Abedi F . Effects of daily consumption of synbiotic bread on insulin metabolism and serum high-sensitivity C-reactive protein among diabetic patients: a double-blind, randomized, controlled clinical trial. Ann Nutr Metab. (2014) 65:34–41. doi: 10.1159/000365153 25196301

[21] Karamali M Dadkhah F Sadrkhanlou M Jamilian M Ahmadi S Tajabadi-Ebrahimi M . Effects of probiotic supplementation on glycaemic control and lipid profiles in gestational diabetes: a randomized, double-blind, placebo-controlled trial. Diabetes Metab. (2016) 42:234–41. doi: 10.1016/j.diabet.2016.04.009, PMID: 27209439

[22] Soleimani A Zarrati Mojarrad M Bahmani F Taghizadeh M Ramezani M Tajabadi-Ebrahimi M . Probiotic supplementation in diabetic hemodialysis patients has beneficial metabolic effects. Kidney Int. (2017) 91:435–42. doi: 10.1016/j.kint.2016.09.040, PMID: 27927601

[23] Xie M Chen P. Influence of bifid-triple viable capsules as adjuvant therapy on beta cell function and insulin-resistance in patients with newly-diagnosed type 2 diabetes mellitus. Chin J Microecol. (2017) 29:454–6.

[24] Kassaian N Feizi A Aminorroaya A Jafari P Ebrahimi MT Amini M. The effects of probiotics and synbiotic supplementation on glucose and insulin metabolism in adults with prediabetes: a double-blind randomized clinical trial. Acta Diabetol. (2018) 55:1019–28. doi: 10.1007/s00592-018-1175-2, PMID: 29931423

[25] Zhang X. Influence of *Bifidobacterium* tetravaccine tables on intestinal flora and pancreaticbetacell function in patients with type 2diabetes mellitus whose blood sugar were not controlled as expected. Chin J Microecol. (2018) 30:1433–6.

[26] Wu X. Regulatory effect of tetragenous viable *bifidobacterium* tablets adjuvant therapy on serum inflammatory factors and oxidative stress in newly diagnosed type 2 diabetic patients. Chin J Microecol. (2020) 32:680–3.

[27] Hasgova T Cao Z Liu D Uggisgulen Z. Application of triple viable bifidobacterium and conventional therapy in elderly type 2 diabetes. Chin J Microecol. (2021) 33:200:1191–5.

[28] Savytska M Kyriienko D Komisarenko I Kovalchuk O Falalyeyeva T Kobyliak N. Probiotic for pancreatic β-cell function in type 2 diabetes: a randomized, double-blinded, placebo-controlled clinical trial. Diabetes Ther. (2023) 14:1915–31. doi: 10.1007/s13300-023-01474-6, PMID: 37713103 PMC10570251

[29] Li L Chen Y Tang Z You Y Guo Y Liao Y. Effect of metformin on gut microbiota imbalance in patients with T2DM, and the value of probiotic supplementation. Allergol Immunopathol. (2024) 52:84–90. doi: 10.15586/aei.v52i4.1101, PMID: 38970270

[30] Savytska M Kyriienko D Zaychenko G Ostapchenko D Falalyeyeva T Kobyliak N. Probiotic co-supplementation with absorbent smectite for pancreatic beta-cell function in type 2 diabetes: a secondary-data analysis of a randomized double-blind controlled trials. Front Endocrinol. (2024) 15:1276642. doi: 10.3389/fendo.2024.1276642, PMID: 38405158 PMC10890794

[31] Tian J Yang Y Yu Z Gao Y Zong X Wu Q . Comparative evaluation of dulaglutide alone vs. dulaglutide combined with probiotics on cardiovascular risk factors in T2DM. Hormones. (2025). doi: 10.1007/s42000-025-00649-z, PMID: 40214965

[32] Page MJ McKenzie JE Bossuyt PM Boutron I Hoffmann TC Mulrow CD . The PRISMA 2020 statement: an updated guideline for reporting systematic reviews. BMJ. (2021) 372:n71. doi: 10.1136/bmj.n71 33782057 PMC8005924

[33] Page MJ Moher D Bossuyt PM Boutron I Hoffmann TC Mulrow CD . PRISMA 2020 explanation and elaboration: updated guidance and exemplars for reporting systematic reviews. BMJ. (2021) 372:n160. doi: 10.1136/bmj.n160, PMID: 33781993 PMC8005925

[34] Higgins J Thomas J Chandler J Cumpston M Li T Page M Cochrane handbook for systematic reviews of interventions version 6.2 The Cochrane Collaboration (2021). Available online at: www.training.cochrane.org/handbook (Accessed April 22, 2025).

[35] American Diabetes Association Professional Practice Committee. 2. Diagnosis and classification of diabetes: standards of care in Diabetes-2025. Diabetes Care. (2025) 48:S27–49. doi: 10.2337/dc25-S002 39651986 PMC11635041

[36] Sweeting A Wong J Murphy HR Ross GP. A clinical update on gestational diabetes mellitus. Endocr Rev. (2022) 43:763–93. doi: 10.1210/endrev/bnac003, PMID: 35041752 PMC9512153

[37] Davidson MB. Historical review of the diagnosis of prediabetes/intermediate hyperglycemia: case for the international criteria. Diabetes Res Clin Pract. (2022) 185:109219. doi: 10.1016/j.diabres.2022.109219, PMID: 35134465

[38] Guyatt G Oxman AD Akl EA Kunz R Vist G Brozek J . GRADE guidelines: 1. Introduction-GRADE evidence profiles and summary of findings tables. J Clin Epidemiol. (2011) 64:383–94. doi: 10.1016/j.jclinepi.2010.04.026, PMID: 21195583

[39] Higgins JP Thompson SG. Quantifying heterogeneity in a meta-analysis. Stat Med. (2002) 21:1539–58. doi: 10.1002/sim.1186, PMID: 12111919

[40] Egger M Davey Smith G Schneider M Minder C. Bias in meta-analysis detected by a simple, graphical test. BMJ. (1997) 315:629–34. doi: 10.1136/bmj.315.7109.629, PMID: 9310563 PMC2127453

[41] Dubey VP Kansagra JJ Sureja VP Kheni DB. Efficacy of a probiotic combination on glycemic index and insulin resistance in adults: a systematic review and meta-analysis. J Diet Suppl. (2025) 22:641–63. doi: 10.1080/19390211.2025.2522463, PMID: 40550754

[42] Sun G Hou H Yang S. The effect of probiotics on gestational diabetes mellitus: an umbrella meta-analysis. BMC Endocr Disord. (2024) 24:253. doi: 10.1186/s12902-024-01751-w, PMID: 39582003 PMC11587629

[43] Singh J Pooja V Kumar P Singh J Dhanda S. Impact of probiotics in alleviating type 2 diabetes risk in clinical trials: a meta-analysis study. Hum Gene. (2023) 35:201149. doi: 10.1016/j.humgen.2023.201149

[44] Markowiak-Kopeć P Śliżewska K. The effect of probiotics on the production of short-chain fatty acids by human intestinal microbiome. Nutrients. (2020) 12:1107. doi: 10.3390/nu12041107, PMID: 32316181 PMC7230973

[45] O’Riordan KJ Collins MK Moloney GM Knox EG Aburto MR Fülling C . Short chain fatty acids: microbial metabolites for gut-brain axis signalling. Mol Cell Endocrinol. (2022) 546:111572. doi: 10.1016/j.mce.2022.111572, PMID: 35066114

[46] Tang R Li L. Modulation of short-chain fatty acids as potential therapy method for type 2 diabetes mellitus. Can J Infect Dis Med Microbiol. (2021) 2021:1–13. doi: 10.1155/2021/6632266, PMID: 33488888 PMC7801078

[47] Xie C Qi C Zhang J Wang W Meng X Aikepaer A . When short-chain fatty acids meet type 2 diabetes mellitus: revealing mechanisms, envisioning therapies. Biochem Pharmacol. (2025) 233:116791. doi: 10.1016/j.bcp.2025.116791, PMID: 39894305

[48] Zhang Y Zhu X Yu X Novák P Gui Q Yin K. Enhancing intestinal barrier efficiency: a novel metabolic diseases therapy. Front Nutr. (2023) 10:1120168. doi: 10.3389/fnut.2023.1120168, PMID: 36937361 PMC10018175

[49] Cristofori F Dargenio VN Dargenio C Miniello VL Barone M Francavilla R. Anti-inflammatory and immunomodulatory effects of probiotics in gut inflammation: a door to the body. Front Immunol. (2021) 12:578386. doi: 10.3389/fimmu.2021.578386, PMID: 33717063 PMC7953067

[50] Ying W Fu W Lee YS Olefsky JM. The role of macrophages in obesity-associated islet inflammation and β-cell abnormalities. Nat Rev Endocrinol. (2020) 16:81–90. doi: 10.1038/s41574-019-0286-3, PMID: 31836875 PMC8315273

[51] Mishra SP Wang S Nagpal R Miller B Singh R Taraphder S . Probiotics and prebiotics for the amelioration of type 1 diabetes: present and future perspectives. Microorganisms. (2019) 7:67. doi: 10.3390/microorganisms7030067, PMID: 30832381 PMC6463158

[52] Mazziotta C Tognon M Martini F Torreggiani E Rotondo JC. Probiotics mechanism of action on immune cells and beneficial effects on human health. Cells. (2023) 12:184. doi: 10.3390/cells12010184, PMID: 36611977 PMC9818925

[53] Boulangé CL Neves AL Chilloux J Nicholson JK Dumas M-E. Impact of the gut microbiota on inflammation, obesity, and metabolic disease. Genome Med. (2016) 8:42. doi: 10.1186/s13073-016-0303-2, PMID: 27098727 PMC4839080

[54] Viana MDM Santos SS Cruz ABO de Jesus MVAC Lauria PSS Lins MP . Probiotics as antioxidant strategy for managing diabetes mellitus and its complications. Antioxidants. (2025) 14:767. doi: 10.3390/antiox14070767, PMID: 40722870 PMC12291856

[55] Elahi D. In praise of the hyperglycemic clamp. A method for assessment of beta-cell sensitivity and insulin resistance. Diabetes Care. (1996) 19:278–86. doi: 10.2337/diacare.19.3.278 8742583

[56] Schupack DA Mars RAT Voelker DH Abeykoon JP Kashyap PC. The promise of the gut microbiome as part of individualized treatment strategies. Nat Rev Gastroenterol Hepatol. (2022) 19:7–25. doi: 10.1038/s41575-021-00499-1, PMID: 34453142 PMC8712374

[57] Manley SE Luzio SD Stratton IM Wallace TM Clark PM. Preanalytical, analytical, and computational factors affect homeostasis model assessment estimates. Diabetes Care. (2008) 31:1877–83. doi: 10.2337/dc08-0097, PMID: 18535197 PMC2518363

[58] Antuna-Puente B Faraj M Karelis AD Garrel D Prud’homme D Rabasa-Lhoret R . HOMA or QUICKI: is it useful to test the reproducibility of formulas? Diabetes Metab. (2008) 34:294–6. doi: 10.1016/j.diabet.2008.02.001 18468934

