# Supplementary figures and images for: Effects of probiotic supplementation on islet β-cell function in subjects with glucose metabolism disorders: a meta-analysis
Source: Front Nutr. 2025 Oct 2;12:1668470. doi: 10.3389/fnut.2025.1668470 (PMC12527896; doi:10.3389/fnut.2025.1668470)

Supplemental Figure 1 Sensitivity analysis limited to RCTs with low risk of bias

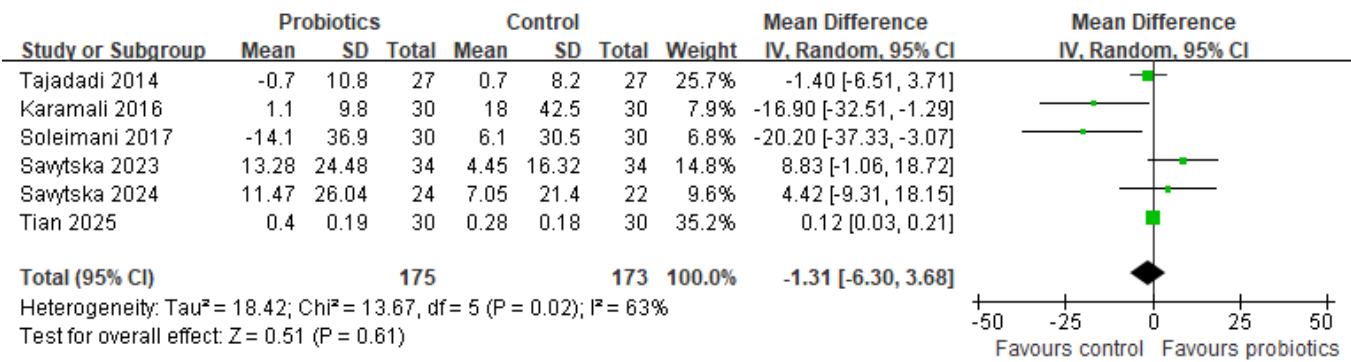

Supplement: Supplementary file 1 [file Image_1.pdf]
